# Mucosal and Systemic Immune Responses to Influenza H7N9 Antigen HA1–2 Co-Delivered Intranasally with Flagellin or Polyethyleneimine in Mice and Chickens

**DOI:** 10.3389/fimmu.2017.00326

**Published:** 2017-04-05

**Authors:** Li Song, Dan Xiong, Hongqin Song, Lili Wu, Meihua Zhang, Xilong Kang, Zhiming Pan, Xinan Jiao

**Affiliations:** ^1^Jiangsu Co-Innovation Center for Prevention and Control of Important Animal Infectious Diseases and Zoonoses, Yangzhou University, Yangzhou, China; ^2^Jiangsu Key Laboratory of Zoonosis, Yangzhou University, Yangzhou, China; ^3^Key Laboratory of Prevention and Control of Biological Hazard Factors (Animal Origin) for Agrifood Safety and Quality, Ministry of Agriculture of China, Yangzhou University, Yangzhou, China; ^4^Joint International Research Laboratory of Agriculture and Agri-Product Safety of the Ministry of Education, Yangzhou University, Yangzhou, China

**Keywords:** avian influenza A (H7N9) virus, hemagglutinin globular head, flagellin, polyethyleneimine, mucosal subunit vaccine

## Abstract

Consecutive cases of human infection with H7N9 influenza viruses since 2013 in China have prompted efforts to develop an effective treatment. Subunit vaccines introduced by intranasal administration can block an infection at its primary site; flagellin (fliC) and polyethyleneimine (PEI) have been shown to be potent adjuvants. We previously generated the hemagglutinin (HA)1–2-fliC fusion protein consisting of the globular head domain (HA1–2; amino acids 62–284) of HA fused with *Salmonella typhimurium* fliC. In the present study, we investigated its effectiveness of both flagellin and PEI as mucosal adjuvants for the H7N9 influenza subunit vaccine. Mice immunized intranasally with HA1–2-fliC and HA1–2-PEI showed higher HA1–2-specific immunoglobulin (Ig)G and IgA titers in serum, nasal wash, and bronchial alveolar lavage fluid. Moreover, splenocyte activation and proliferation and the number of HA1–2-specific interferon (IFN)-γ- and interleukin (IL)-4-producing splenocytes were markedly increased in the fliC and PEI groups; in the latter, there were more cells secreting IL-4 than IFN-γ, suggesting that fliC induced T helper type (Th)1 and Th2 immune responses, and PEI induced Th2-biased responses, consistent with the serum antibody isotype pattern (IgG1/IgG2a ratio). Furthermore, virus challenge was performed in a chicken model. The results showed that chickens receiving fliC and PEI adjuvant vaccine exhibited robust immune responses leading to a significant reduction in viral loads of throat and cloaca compared to chickens receiving only HA1–2. These findings provide a basis for the development of H7N9 influenza HA1–2 mucosal subunit vaccines.

## Introduction

In the spring of 2013, an H7N9 subtype of avian influenza virus infecting humans was discovered in China; although initially the virus replicated silently in chickens without causing disease (1), rapid development throughout the country led to an outbreak. Despite a lot of work to control the infection, H7N9 influenza viruses have continued to spread, leading to human infections in many provinces of inland China, Taiwan, and Hong Kong, with 212 of the 571 confirmed cases proving fatal as of February 23, 2015 (2). To date, there exist no licensed vaccines for H7N9 infection. Therefore, it is critical to develop a vaccination strategy to protect against H7N9 influenza.

Vaccination is one of the most efficacious and cost-effective medical interventions. The advantage of subunit vaccines is that they provide a safe and specific stimulus for the induction of immunity (3). The administration of antigen to mucosal surfaces is possibly the best method of inducing mucosal immune responses at distant as well as local sites (4), and efforts are on the way to develop mucosal vaccines for influenza (5, 6). A main obstacle to mucosal subunit vaccines has been the lack of an appropriate adjuvant; the only one licensed for human use (aluminum) is unsuitable for mucosal applications (3).

Toll-like receptors (TLRs) are a family of receptors that recognize pathogen-associated molecular patterns on cells of the innate immune system and play a key role in responding and determining to microbial infections. Molecular adjuvants based on TLR ligands have been shown to enhance the immunogenicity of vaccines, and these ligands are increasingly recognized as key adjuvant targets (7). Flagellin, a TLR5 ligand, is also known to exhibit potent adjuvant effects on inducing immune response (8, 9). Our previous study showed that hemagglutinin (HA)1–2 of H7N9 influenza virus fused to *Salmonella typhimurium* fliC induced robust immune responses in mice immunized intraperitoneally (10, 11). Influenza subunit vaccines based on HA1–2 and flagellin have been shown to exert protective effects in other studies (12, 13), suggesting that HA1–2 is a promising subunit vaccine candidate. However, in these studies, mice were immunized subcutaneously or intraperitoneally; there are few reports describing the use of flagellin as a mucosal adjuvant in influenza subunit vaccines (14).

A recent study found that polyethyleneimine (PEI) has potent mucosal adjuvant activity for viral subunit soluble glycoprotein antigens, including gp140 derived from human immunodeficiency virus 1 and HA protein from influenza virus (15). We speculated that intranasal immunization with PEI combined with HA1–2 of H7N9 influenza virus could improve mucosal and systemic immunity.

In this study, we used fliC and PEI as mucosal adjuvants for H7N9 influenza HA1–2 subunit vaccine, with cholera toxin B subunit (CTB) used as a positive control. HA1–2-fliC and HA1–2-PEI increased immunoglobulin (Ig)G and IgA production in serum, nasal wash, and bronchial alveolar lavage fluid (BALF) as well as the number of HA1–2-specific interferon (IFN)-γ- and interleukin (IL)-4-producing splenocytes. Mice vaccinated intranasally with candidate adjuvant-based influenza subunit vaccines developed rapid systemic and robust local mucosal immune responses. In addition, chickens receiving flagellin and PEI adjuvant candidate vaccines exhibited robust immune responses with decreased viral loads in throat and cloaca following H7N9 influenza virus challenge.

## Materials and Methods

### Ethics Statement

Female C3H/HeJ mice (a spontaneous mutation in TLR4 gene) aged 6 weeks were purchased from the SLAC Laboratory Animal Co. Ltd., Shanghai, China. We used C3H/HeJ mice as a model ruling out the role for TLR4 responses in the adjuvant activity. Two-week-old specific-pathogen-free (SPF) White Leghorn chickens were purchased from poultry institute, Shandong academy of agricultural science. All mice and birds were housed in isolators and kept in a room with controlled temperature, light, and ventilation. Pathogen-free water and diet were supplied *ad libitum*. All animal studies were performed in accordance with the Committee on the Ethics of Animal Experiments of Yangzhou University (Approval ID: SYXK [Su] 2012–0029).

### Viruses

The avian influenza H7N9 virus (A/chicken/Jiangsu/CZT4/2013) used in this study was provided by the Animal Infectious Disease Laboratory of Yangzhou University. The inactivated virus using 0.1% formalin was used as hemagglutination inhibition (HAI) antigen, and the live virus was used in the viral challenge experiment.

### Preparation of Candidate Vaccines

Recombinant His-tagged HA1–2 and HA1–2-fliC proteins were prepared as previously described (10). HA1–2-PEI candidate vaccine was prepared at least 2 h before vaccination by mixing the antigen to a pre-diluted PEI solution (25-kDa branched form; Sigma-Aldrich, St. Louis, MO, USA) (11). CTB adjuvant (Sigma-Aldrich) was mixed with purified HA1–2 protein just prior to immunization.

### Nasal Vaccination and Sampling

**Figure 1:**
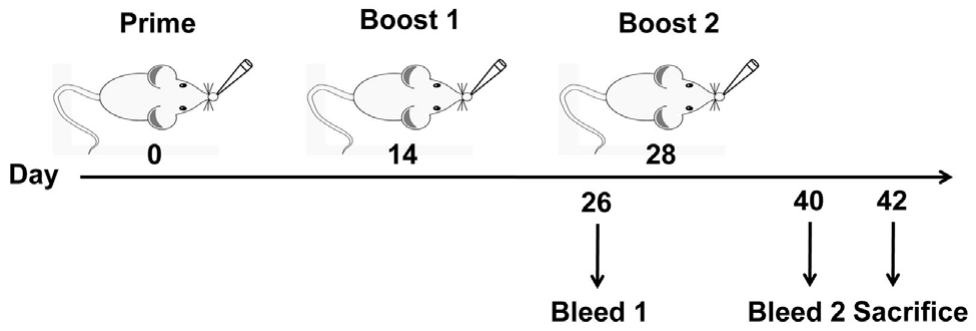
**Vaccination and blood sample collection schedule**. C3H/HeJ mice (*n* = 6 per group) were vaccinated intranasally with three doses of PBS, HA1–2, HA1–2-fliC, or HA1–2 combined with PEI or CTB on days 0, 14, and 28. Animals were bled on days 26 and 40 following the second and third immunizations. Resultant serum samples were used to assess serum antibody responses, and all mice were sacrificed on day 42 to evaluate cellular immune responses.

C3H/HeJ mice (*n* = 6) were lightly anesthetized with Zoletil (Virbac, Carros, France) (10 mg/kg body weight) and intranasally vaccinated with a volume of 50 μl containing 10 μg HA1–2, 30 μg HA1–2-fliC (containing 10 μg HA1–2), 10 μg HA1–2 mixed with 20 μg PEI, 20 μg CTB in phosphate-buffered saline (PBS), or 50 μl PBS, respectively, on days 0, 14, and 28. The volume of 50 μl was administered dropwise to external nares of the mice (25 μl per nostril) using a micropipette. Animals were bled 12 days after the second and third immunizations. Serum samples were analyzed by HAI assay and enzyme-linked immunosorbent assay (ELISA) to detect the HA1–2-specific IgA and IgG titers and its subtypes (IgG1 and IgG2a). Two weeks after the last immunization, nasal wash and BALF were collected by washing the organs three times with 0.2 or 0.5 ml sterile PBS, respectively, and secretory IgA and IgG levels were determined by ELISA (Figure 1).

### Enzyme-Linked Immunosorbent Assay

Antigen-specific IgG, IgG1, IgG2a, and IgA in serum and IgA and IgG in lavage fluid were detected by indirect ELISA as previously described (10). Briefly, 96-well plates were coated with 2.5 μg/ml glutathione S-transferase-tagged HA1–2 antigen in 50 mM carbonate buffer (pH 9.6) at 4°C overnight. After washing and blocking, samples at twofold dilution were added for 2 h at 37°C. Horseradish peroxidase-conjugated goat anti-mouse IgA (1:2,000), IgG (1:10,000), IgG1 (1:3,000), or IgG2a (1:3,000) (Invitrogen, Carlsbad, CA, USA) was added by incubation for 1 h at 37°C, with 3, 3′, 5, 5′-tetramethybenzidine used as a substrate. The reaction was terminated with 2 M H_2_SO_4_, and the absorbance at 450 nm was read on a microplate reader (BioTek, Winooski, VT, USA). The cutoff value was defined as the mean + 2 SD of negative control. The antibody titers were defined as the reciprocal of the highest dilution of samples that had a reading above the cutoff value.

### HAI Assay

Animals were bled 12 days after the third immunizations. Serum samples were analyzed by HAI assay, which were conducted according to our previously described procedure (10).

### Isolation of Splenic Lymphocytes

Two weeks after the third immunization, splenic lymphocytes were obtained from the spleens of mice by density gradient centrifugation using Lymphoprep (specific gravity 1.077) (Sigma-Aldrich) according to the manufacturer’s instructions. Single-splenocyte suspensions were prepared in complete Roswell Park Memorial Institute (RPMI) 1640 medium plus 1% penicillin-streptomycin/l-glutamine and 10% fetal bovine serum (Gibco, Carlsbad, CA, USA) at a final concentration of 2 × 10^6^ cells/ml.

### Cell Proliferation ELISA Based on Bromo-2′-Deoxyuridine (BrdU)

Splenic lymphocytes were obtained from each mouse after the third vaccination as described above, and the cell proliferation assay of splenic lymphocyte pre-treated with 10 μg/ml of HA1–2 was performed using the commercially available ELISA Kit based on 5-BrdU (Roche Diagnostics, Tokyo, Japan) according to the manufacturer’s protocol.

### IFN-γ and IL-4 Enzyme-Linked Immunospot (ELISPOT) Assays

Splenic lymphocytes were obtained from mice after the third vaccination, and the single-splenocyte suspensions were prepared for quantifying HA1–2-specific IFN-γ- or IL-4-producing cells using the BD ELISPOT set (BD Biosciences, Franklin Lakes, NJ, USA). All ELISPOT assays were performed according to our previously described procedure (11).

### Histological Analysis

Groups of C3H/HeJ mice (*n* = 3) were anesthetized with Zoletil (Virbac) and vaccinated intranasally with one dose of the candidate vaccines. At 12-h postvaccination, the mice from each group were sacrificed, and the left lungs and trachea were removed and fixed in 10% neutral formalin at room temperature for 48 h. Serial tissue sections (4 μm thick) were prepared and stained with hematoxylin and eosin (H&E) for histological analysis.

### Chicken Vaccination and Viral Challenge

A total of 70 SPF chickens at 2-week-old were randomized into 5 groups (*n* = 14). Prior to challenge, all birds were immunized, and the immunization dosage and programs were the same as mice vaccination. In each group, the birds were immunized intranasally with a final volume of 100 μl/dose/chicken candidate vaccines on days 0, 14, and 28. At 2 weeks after the third immunization (on day 42), chickens (*n* = 6) in each group were bled from the wing vein and sacrificed for immune response determination. The others (*n* = 8) were inoculated intranasally with 10^6^ 50% egg infectious dose (EID_50_) of H7N9 influenza virus in a 200 μl volume. The throat and cloaca swabs were collected from chickens at 3, 5, and 7 day postinoculation (dpi) and resuspended in 1 ml PBS for viral RNA extraction followed by real-time PCR (RT-PCR) for quantitative analysis of virus.

### Quantitative Analysis of Viral Load

Viral RNA was extracted from the swab samples of chicken using TIANamp Virus RNA Kit (TianGen, Beijing, China). cDNA was synthesized from mRNA using a PrimeScrip RT reagent Kit (TaKaRa, Dalian, China) according to the manufacturer’s instructions. For viral quantification, the primers forward: 5′-GGAGTTCTAATTATCAACAATC-3′; reverse: 5′-TCCCATAGATTTTCCTCTC-3′; and the TaqMan probe: 5′-(FAM) CCAGGAGCGAGACCACAAGTTA (TAMRA)-3′ were designed to amplify and detect a 185-base pair segment of HA gene of H7N9 influenza virus. The reaction was run on an ABI 7500 with the following steps: 95°C for 30 s, followed by 40 cycles of 95°C for 5 s and 60°C for 34 s. Data analyses were performed using the 7500 software supplied with the instrument. The copy number of the HA gene was calculated on the basis of a standard curve using an HA-containing plasmid pCold-HA1–2 (10) of known concentration as a standard. The copy numbers of the HA gene were quantified by the following equation: copies/μl = [(6.02 × 10^23^ copies/mol) × concentration of plasmid g/μl]/(number of base pairs of the plasmid × 2 × 324.5).

### Statistical Analysis

Data are expressed as mean ± SEM unless otherwise stated. The differences between groups were analyzed using Mann–Whitney’s *U* test with a 95% confidence interval (SPSS 16.0). *P* < 0.05 was regarded as significant.

## Results

### Antibody Response in Serum

**Figure 2:**
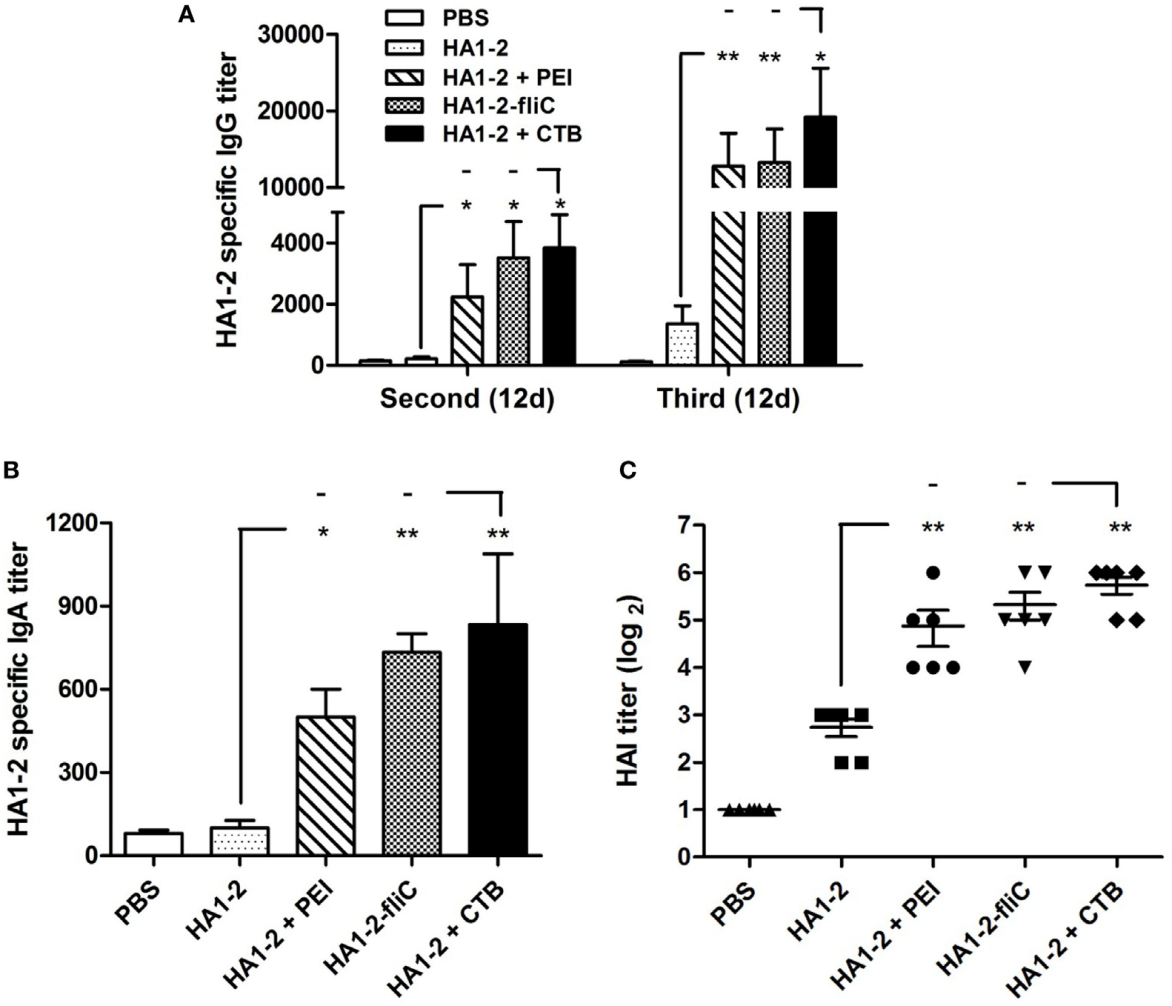
**Antibody response in serum**. C3H/HeJ mice (*n* = 6 per group) were vaccinated intranasally with three doses of HA1–2, HA1–2-fliC, PBS, or HA1–2 combined with PEI or CTB. Animals were bled 12 days after the second and third immunizations. Antibody titers were measured by ELISA or hemagglutination inhibition (HAI) assay. **(A)** HA1–2-specific IgG titers after the second and third immunizations. **(B)** HA1–2-specific IgA titers after the third immunizations. **(C)** HAI titers after the third immunizations.

Mice were vaccinated intranasally with HA1–2, HA1–2-fliC, or HA1–2 combined with PEI or CTB on days 0, 14, and 28 (Figure 1). The capacity of these candidate vaccines to induce a humoral immune response was evaluated by detecting the presence of HA1–2-specific antibodies in the serum by ELISA at 12 days post the second and third vaccination. HA1–2-specific IgG titers were higher after the third as compared to after the second immunization (>10,000 vs. ≈3,000). HA1–2-fliC and HA1–2-PEI elicited higher IgG titers than HA1–2 alone (Figure 2A). Similarly, HA1–2-specific IgA titers in serum induced by HA1–2-fliC and HA1–2-PEI (340 and 480 on average, respectively) after the third immunization were higher than that of HA1–2 alone (66.6 on average) (Figure 2B).

In addition, HAI titers in the adjuvant groups following the third immunization were about fourfold to sixfold higher than in animals immunized with HA1–2 antigen alone (29.3 for PEI, 40 for HA1–2-fliC, and 6.6 for HA1–2) (Figure 2C). The fliC and PEI adjuvant group elicited superior immune responses and induced similar HAI titers compared to the positive control.

### HA1–2-Specific IgG Subtype Responses to Candidate Vaccines

**Figure 3:**
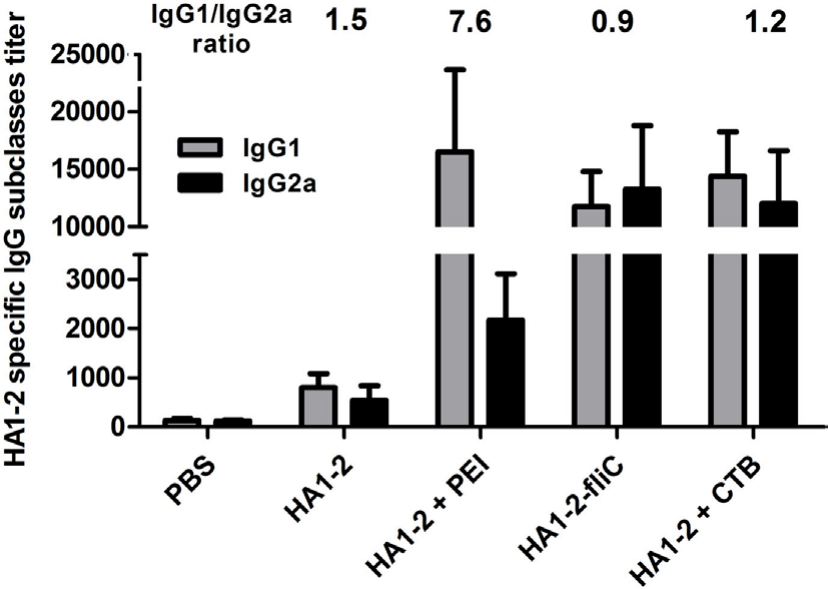
**HA1–2-specific IgG subtype responses to candidate vaccines**. C3H/HeJ mice (*n* = 6 per group) were vaccinated intranasally with three doses of HA1–2, HA1–2-fliC, PBS, or HA1–2 combined with PEI or CTB. Animals were bled 12 days after the third immunization and HA1–2-specific IgG subtype (IgG1 and IgG2a) titers were measured by ELISA.

The IgG subtype (IgG1 and IgG2a) induced in each group was assessed by ELISA, and the IgG1/IgG2a ratio was calculated. A ratio of 0.5 or less indicates a T helper type (Th)1-biased response. A ratio of 2.0 or more indicates a Th2-biased response. Ratios between 0.5 and 2.0 indicate a mixed or balanced response (16). In the group immunized with PEI-supplemented vaccine, the IgG1 titer was significantly higher than the IgG2a titer (*P* < 0.05). The highest IgG1/IgG2a ratio (7.6) was observed in the group immunized with PEI-supplemented vaccine, while the ratio was lower in the HA1–2-fliC group (0.9) and similar to that observed for CTB (1.2) vaccine and HA1–2 alone (1.5) (Figure 3).

### Antibody Response in Nasal Wash and BALF

**Figure 4:**
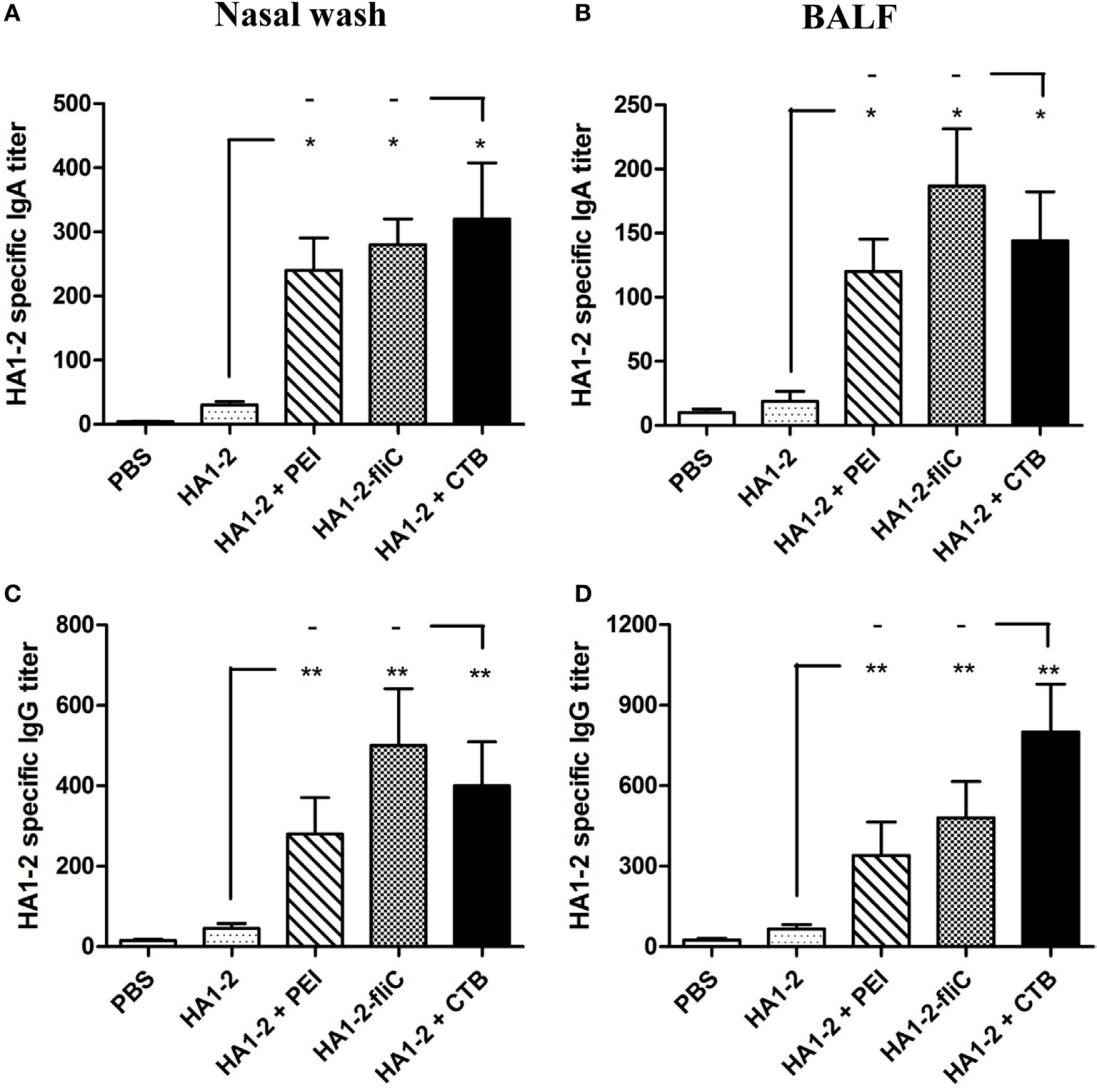
**Antibody responses in nasal wash and bronchial alveolar lavage fluid (BALF)**. HA1–2 specific IgG and IgA titers were induced by intranasal immunization with candidate vaccines. **(A–D)** Nasal wash and BALF samples were collected 2 weeks after the last immunization, and HA1–2-specific IgA and IgG titers in nasal wash **(A,C)** and BALF **(B,D)** of immunized mice (*n* = 6) were measured by ELISA.

The amount of IgA secreted by the mucosa is used as an index of mucosal immune response (17). To determine whether PEI and fliC are effective adjuvants in the mucosal immune response, nasal wash and BALF of mice immunized three times with candidate vaccines were obtained by three successive PBS washes, and HA1–2-specific IgA and IgG levels were determined by ELISA. Compared to HA1–2 alone, HA1–2-specific IgA as well as IgG levels in the nasal wash and BALF were increased in HA1–2-PEI and HA1–2-fliC groups (Figure 4). HA1–2-fliC immunization induced higher HA1–2-specific nasal and BAL mucosal IgA titers (280, *P* < 0.05 and 187, *P* < 0.05, respectively) than HA1–2 immunization (30 and 19 on average, respectively). Similarly, HA1–2-PEI stimulated nasal and BAL mucosa to induce higher IgA titers (240, *P* < 0.05 and 120, *P* < 0.05, respectively) (Figures 4A,B). Moreover, nasal and BAL mucosal IgG titers induced by HA1–2-fliC (500, *P* < 0.01 and 480, *P* < 0.01, respectively) and HA1–2-PEI (280, *P* < 0.01 and 340, *P* < 0.01, respectively) were also significantly higher than HA1–2 immunization (46 and 67 on average, respectively) (Figures 4C,D).

### Cellular Immune Response in Spleen

**Figure 5:**
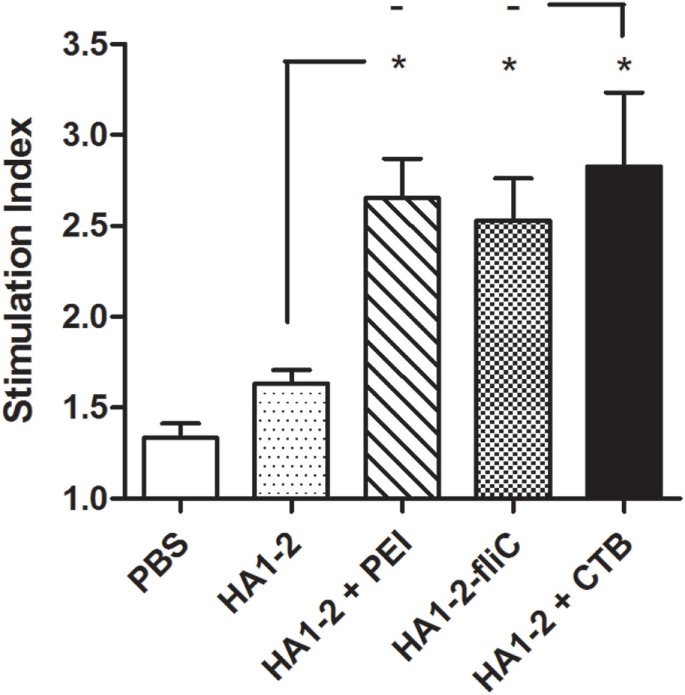
**Splenocyte stimulation index (SI)**. Splenocytes were prepared from the spleens of mice after the third immunization, and the SI of cells in response to purified HA1–2 (10 μg/ml) protein was calculated based on cell proliferation, as determined with the BrdU assay, using the following equation: SI = (OD_450_ − OD_690_ of antigen-treated cells)/(OD_450_ − OD_690_ of untreated cells).

All vaccinated mice were euthanized 14 days after the third immunization. Splenic lymphocytes were isolated from vaccinated mice and stimulated with HA1–2 for 48 h, and the cellular immune response was assessed with the BrdU assay. Compared to the response of mice treated with HA1–2 alone (1.6 on average), the fliC- and PEI-supplemented groups showed significantly higher stimulation index (2.5, *P* < 0.05 and 2.7, *P* < 0.05, respectively) (Figure 5).

### IFN-γ- and IL-4-Producing Cells Induced by Candidate Vaccines

**Figure 6:**
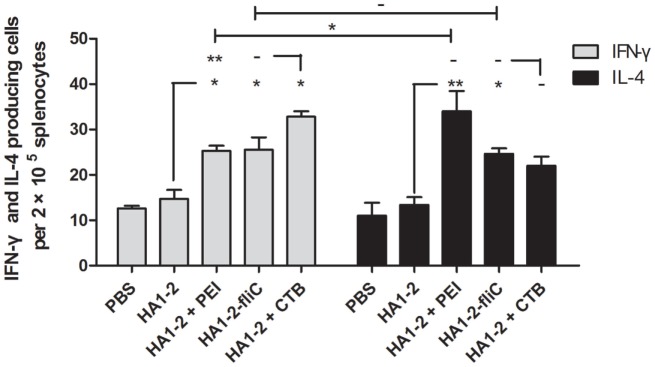
**Quantitative analysis of interferon (IFN)-γ- and interleukin (IL)-4-producing cells**. On day 42, mice (*n* = 6) were euthanized and single-cell suspensions prepared from the spleens were cultured for 48 h, then stimulated with purified protein HA1–2 (5 μg/ml). IFN-γ and IL-4 secretion was detected in triplicate with the ELISPOT assay.

To further evaluate the ability of vaccine candidates to induce cellular immune responses and to estimate the immune types, ELISPOT assays were performed to determine the numbers of cells secreting IFN-γ and IL-4. Splenic lymphocytes were prepared 2 weeks after the third immunization and stimulated with HA1–2 *in vitro*. The average numbers of IFN-γ- and IL-4-producing cells were higher in HA1–2-fliC (26, *P* < 0.05 and 25, *P* < 0.05, respectively) and PEI-supplemented (25, *P* < 0.05 and 34, *P* < 0.01, respectively) vaccine groups than in the HA1–2 vaccine group (14 and 13, respectively). Notably, a significantly greater number of IL-4- than IFN-γ-producing cells were induced by PEI-supplemented vaccine (*P* < 0.05) (Figure 6), indicating that Th2-biased immune responses were induced by PEI-supplemented vaccine. In contrast, equal numbers of IFN-γ- and IL-4-producing cells were observed in the HA1–2-fliC group, indicating that both Th1- and Th2-associated immune responses were induced by the vaccination.

### Histological Analysis of Trachea and Lung

**Figure 7:**
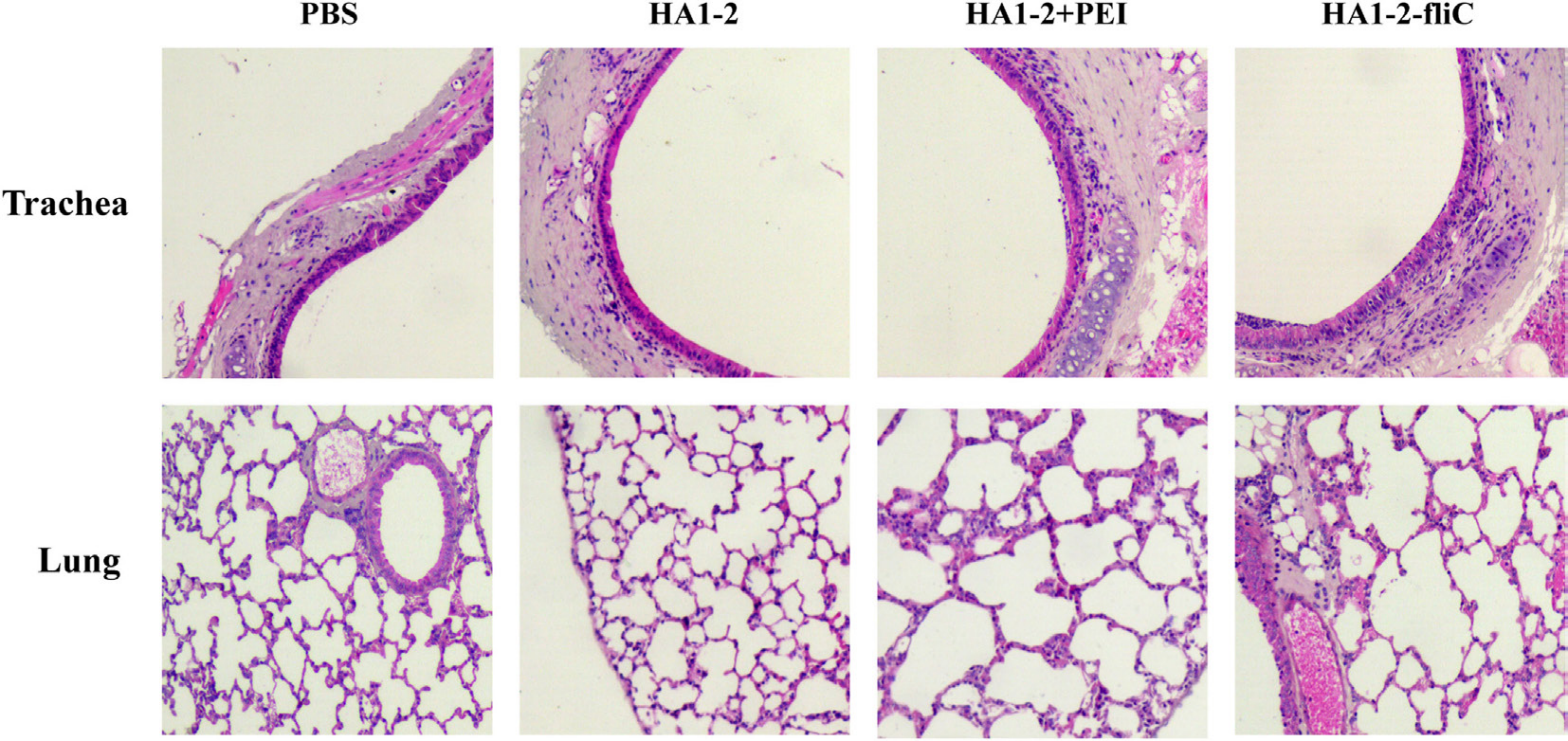
**Histological analysis**. C3H/HeJ mice (*n* = 3) were vaccinated intranasally with one dose of PBS, HA1–2, HA1–2-fliC, or HA1–2 combined with PEI. The lungs and trachea were removed from mice at 12-h postvaccination, and tissue sections were prepared and stained with H&E for histological analysis (100× magnification).

Formalin-fixed, paraffin-embedded lung and trachea sections from groups of three mice were prepared at 12 h and stained with H&E. The intact structures were observed in trachea and lung sections from all groups of mice vaccinated with candidate vaccines or PBS. No evidence of any tissue damage was observed. Moreover, sections of trachea and lung from mice vaccinated with HA1–2-fliC and HA1–2-PEI showed activated immune responses with a number of neutrophils around the trachea or into the tracheal cavity compared to control sections (Figure 7).

### Virus Loads in Throat and Cloaca of H7N9 Virus Challenged Chickens

**Figure 8:**
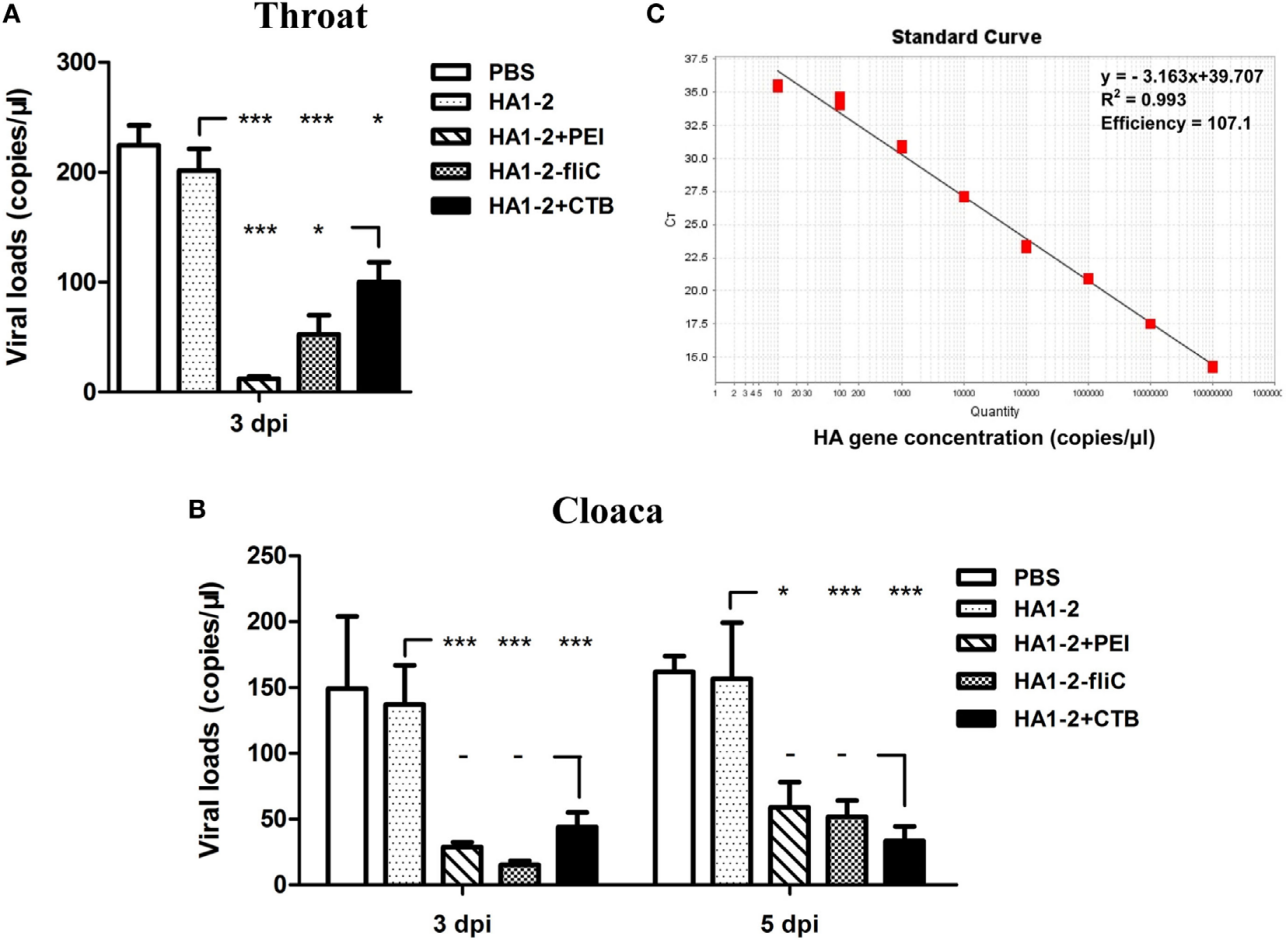
**Viral loads detection in throat and cloaca following H7N9 virus challenge of vaccinated chickens**. Two-week-old SPF chickens (*n* = 8) were inoculated intranasally with 10^6^ EID_50_ of H7N9 influenza virus in a 200 μl volume. The throat and cloaca swabs were collected from chickens and resuspended in 1 ml PBS for viral RNA extraction followed by real-time PCR (RT-PCR) for quantitative analysis of virus. **(A)** Viral loads in throat following H7N9 virus challenge at 3 dpi. **(B)** Viral loads in cloaca following H7N9 virus challenge at 3 and 5 dpi. **(C)** Standard curves (based on plasmid DNA) indicating the linearity and efficiency for detecting HA by RT-PCR.

Prior to challenge, the results showed that chickens vaccinated with HA1–2-fliC and HA1–2-PEI could elicit significantly increased serum IgG, nasal wash and BALF IgA titers compared to that vaccinated with HA1–2 (Figure S1 in Supplementary Material). In order to investigate whether flagellin and PEI could improve the protective efficacy of HA1–2, we detected the viral loads in throat and cloaca of H7N9 virus challenged chickens. The copy numbers of the HA gene were determined using RT-PCR with an HA-containing plasmid of known concentrations (Figure 8C). At 3 dpi, the average virus loads in throat and cloaca of chickens vaccinated with HA1–2-fliC were significantly reduced (52, *P* < 0.001, and 15, *P* < 0.001, respectively) compared to chickens vaccinated with HA1–2 (225 and 137, respectively). Similarly, the average levels of virus were also significantly decreased in PEI-vaccinated chickens (12, *P* < 0.001, and 28, *P* < 0.001, respectively). At 5 dpi, significantly lower virus loads were observed in cloaca in HA1–2-fliC (51, *P* < 0.001) and PEI-adjuvant group (58, *P* < 0.05) compared to chickens vaccinated with HA1–2 (157 on average) (Figures 8A,B).

## Discussion

Inactivated vaccines are typically administered *via* the subcutaneous or intramuscular route to prevent influenza virus infection (18, 19). Although these vaccines induce serum IgG antibodies, they induce poor IgA at respiratory mucosal sites. In addition, an intranasal vaccine would be easier to administer than an intramuscular vaccine and could have fewer adverse effects, thereby more people may be willing to be vaccinated (20, 21). This study was carried out in order to improve the immunogenicity of a nasally administered influenza HA1–2 subunit vaccine that would induce both systemic and mucosal antibody responses.

To determine the capacity of candidate vaccines to induce humoral immunity, we measured HA1–2-specific antibody responses in serum. Higher IgA and IgG titers were detected in the HA1–2-fliC and HA1–2-PEI than in the HA1–2 group, which were similar to those observed by intraperitoneal immunization (10). The serum IgA titer was less robust but was elevated compared to HA1–2 without adjuvant. It has been reported that serum HAI titers are correlated with the degree of protection conferred by inactivated influenza vaccines administered intramuscularly or subcutaneously, and they therefore serve as the standard for evaluating these vaccines in adults (22, 23). We evaluated the ability of serum IgG antibodies to neutralize the influenza virus in supplemented vaccine groups and found that all animals in these groups had higher HAI titers (1:40) than the HA1–2 group.

IgA provides the greatest immunological defense against microbial infection at the mucosal surface and is considered an important indicator of mucosal immunity (16). Since the nasal cavity and bronchial alveoli comprise a large surface area of mucous membrane, IgA and IgG levels were measured in nasal wash and BALF 2 weeks after the last vaccination. IgG titers were increased at the mucosal surface, and IgA levels were higher in mice vaccinated with HA1–2-fliC and HA1–2-PEI than in those without adjuvant treatment. Additionally, previous study has showed that intranasal administration of influenza vaccines induces higher virus-specific IgA and IgG2a responses in mice than that by subcutaneous administration (24), which emphasized the importance of intranasal route in vaccination against influenza.

The ideal vaccine stimulates serum IgG and mucosal IgA production, a cytokine response, and a longer-lasting immune response (25). We found that mice vaccinated with HA1–2-fliC and HA1–2-PEI developed a rapid systemic immune response in serum and robust mucosal immune response in the nasal cavity and bronchial alveoli. T cells play an important role in protection against various strains of influenza virus (26). We evaluated proliferation and cytokine secretion in splenocytes following HA1–2 stimulation *in vitro*. As expected, cell proliferation was increased, which was consistent with previous study showing that a significant increase of PMBC proliferation was observed in chickens vaccinated with mFliC or pFliC-adjuvanted vaccines (27), yielding higher numbers of IFN-γ- and IL-4-producing cells in the adjuvant groups than in mice administered the HA1–2 vaccine, suggesting that antigen-specific lymphocytes were activated in the spleen.

Cluster of differentiation 4-positive Th differentiates into Th1 and Th2 subtypes, which are associated with primarily cellular immune responses and antibody production, respectively (28). HA1–2-fliC and PEI-supplemented vaccines induced higher numbers of spleen cells to secrete IL-4 and IFN-γ, whereas PEI induced more cells to secrete IL-4. In mice, IFN-γ and IL-4 are associated with Th1 and Th2 responses, respectively (25, 29). Thus, our data indicate that mice vaccinated with HA1–2-fliC exhibited a balanced Th1/Th2 response, and HA1–2-PEI exhibited a Th2-biased response. These results reflected the pattern of antibody isotypes (IgG1/IgG2a); HA1–2-fliC induced a high level of IgG1 and IgG2a antibodies, and the IgG1/IgG2a ratio was close to 1, indicating a balanced Th1/Th2-type immune response. This is in contrast to another study reporting that co-delivery of fliC with an antigen induced a Th2 response in mice (30). We speculate that mouse strain difference affect the immune response bias. It was recently reported that systemic delivery of branched PEI stimulated a mixed Th1/Th2-type adaptive immune response (31). In our study, the third dose of the HA1–2-PEI vaccine boosted serum IgG2a and IgG1 titers, producing a IgG1/IgG2a ratio ≥2.0, which indicated a Th2-biased immune response.

Mucosal vaccines have advantages for respiratory diseases, but one of the challenges is to identify a safe vehicle for intranasal delivery of influenza antigens to induce protective mucosal and serum antibody responses (32). In our study, the cationic polymer PEI was used to overcome barriers to intranasal administration. We found that PEI could be used as an adjuvant for a mucosal influenza vaccine when administered intranasally with influenza HA1–2 antigen. PEI was recently reported to stimulate mixed T-cell responses in mucosa (15, 33) and could be modified to target dendritic cells, which are professional antigen-presenting cells that express high levels of surface mannose receptor (29). Additionally, safety evaluation of HA1–2-fliC and HA1–2-PEI candidate vaccines was performed in this study. No tissue damages of lung and trachea were observed in vaccinated mice. A number of neutrophils appeared around the trachea or into the tracheal cavity from mice vaccinated with HA1–2-fliC and HA1–2-PEI, which was consistent with the results of other studies that flagellin and PEI could induce innate immunity as vaccine adjuvants (15, 34).

In this study, significantly enhanced mucosal and systemic immune responses were induced by chickens vaccinated with HA1–2-fliC and HA1–2-PEI. These results were consistent with the study of flagellin used as an adjuvant in chickens immunized intramuscularly or intranasally with H5N2 influenza vaccines (27). Following H7N9 virus challenge, chickens vaccinated with adjuvant-based HA1–2 vaccines exhibited significantly reduced viral loads in throat and cloaca compared to that vaccinated with HA1–2 vaccine. Similar results were observed in cloaca of chickens challenged by low pathogenic H9N2 influenza virus (35). Collectively, the presence of flagellin and PEI could induce antigen-specific immune responses that are capable of increasing virus clearance in throat and cloaca of chickens.

Adjuvant system (AS)04 was recently licensed for human use and has been successfully used with recombinant human papillomavirus vaccine (36–38). AS04 is a mixed adjuvant of aluminum salt and the TLR4 agonist monophosphoryl lipid A that triggers innate immune cytokine responses and thereby enhances adaptive immunity (39). Based on this principle, the present study demonstrated that PEI was an effective adjuvant that increased the immunogenicity of influenza HA1–2 vaccine for intranasal immunization. Future studies will assess the potential effects of combined adjuvants such as alum and TLR ligands (e.g., fliC) or PEI.

In summary, our results indicate that both fliC and PEI could improve the potency of HA1–2-based nasal vaccines. Their intranasal immunization elicited enhanced humoral and cellular as well as local mucosal immune responses in mouse and chicken models. In addition, challenge-protection study showed that chickens receiving fliC and PEI adjuvant vaccines exhibited robust immune responses leading to a significant reduction in viral loads of throat and cloaca. These findings provide a basis for investigating mucosal immune responses as well as potential adjuvants for use with H7N9 influenza mucosal vaccines.

## Author Contributions

XJ, ZP, and LS designed the research; LS, DX, LW, and MZ performed the experiments; LS, DX, HS, and XK analyzed the data; LS, XJ, ZP, and DX participated in writing the paper. All authors reviewed the manuscript.

## Conflict of Interest Statement

The authors declare that the research was conducted in the absence of any commercial or financial relationships that could be construed as a potential conflict of interest.

## References

[1] Chen Y Liang WF Yang SG Wu NP Gao HN Sheng JF Human infections with the emerging avian influenza A H7N9 virus from wet market poultry: clinical analysis and characterisation of viral genome. Lancet (2013) 381:1916–25. 10.1016/S0140-6736(13)60903-4 23623390 PMC7134567

[2] World Health Organization. Data from: Human Cases of Avian Influenza A(H7N9) Virus Infection to Date. (2015). Available from: http://www.who.int/influenza/human_animal_interface/influenza_h7n9/RiskAssessment_H7N9_23Feb20115.pdf?ua=1&ua=1

[3] McKenzie BS Brady JL Lew AM. Mucosal immunity: overcoming the barrier for induction of proximal responses. Immunol Res (2004) 30:35–71. 10.1385/IR:30:1:035 15258310

[4] Lehner T Bergmeier L Wang Y Tao L Mitchell E A rational basis for mucosal vaccination against HIV infection. Immunol Rev (1999) 170:183–96. 10.1111/j.1600-065X.1999.tb01338.x 10566151

[5] Major D Chichester JA Pathirana RD Guilfoyle K Shoji Y Guzman CA Intranasal vaccination with a plant-derived H5 HA vaccine protects mice and ferrets against highly pathogenic avian influenza virus challenge. Hum Vaccin Immunother (2015) 11:1235–43. 10.4161/21645515.2014.988554 25714901 PMC4514375

[6] Kim E-D Han SJ Byun Y-H Yoon SC Choi KS Seong BL Inactivated eyedrop influenza vaccine adjuvanted with poly(I:C) is safe and effective for inducing protective systemic and mucosal immunity. PLoS One (2015) 10:e0137608. 10.1371/journal.pone.0137608 26355295 PMC4565664

[7] Steinhagen F Kinjo T Bode C Klinman DM. TLR-based immune adjuvants. Vaccine (2011) 29:3341–55. 10.1016/j.vaccine.2010.08.002 20713100 PMC3000864

[8] Liu G Song L Reiserova L Trivedi U Li H Liu X Flagellin-HA vaccines protect ferrets and mice against H5N1 highly pathogenic avian influenza virus (HPAIV) infections. Vaccine (2012) 30:6833–8. 10.1016/j.vaccine.2012.09.013 23000130

[9] Liu G Tarbet B Song L Reiserova L Weaver B Chen Y Immunogenicity and efficacy of flagellin-fused vaccine candidates targeting 2009 pandemic H1N1 influenza in mice. PLoS One (2011) 6:e20928. 10.1371/journal.pone.0020928 21687743 PMC3110246

[10] Song L Xiong D Kang XL Yang Y Wang J Guo YX An avian influenza A (H7N9) virus vaccine candidate based on the fusion protein of hemagglutinin globular head and *Salmonella typhimurium* flagellin. BMC Biotechnol (2015) 15:79. 10.1186/s12896-015-0195-z 26286143 PMC4544785

[11] Song L Xiong D Hu MZ Kang XL Pan ZM Jiao XA. Immunopotentiation of different adjuvants on humoral and cellular immune responses induced by HA1-2 subunit vaccines of H7N9 influenza in mice. PLoS One (2016) 11:e0150678. 10.1371/journal.pone.0150678 26930068 PMC4773109

[12] Son LZ Nakaar V Kavita U Price A Huleatt J Tang J Efficacious recombinant influenza vaccines produced by high yield bacterial expression: a solution to global pandemic and seasonal needs. PLoS One (2008) 3:e2257. 10.1371/journal.pone.0002257 18493310 PMC2373928

[13] Song LZ Zhang Y Yun NE Poussard AL Smith JN Smith JK Superior efficacy of a recombinant flagellin: H5N1 HA globular head vaccine is determined by the placement of the globular head within flagellin. Vaccine (2009) 27:5875–84. 10.1016/j.vaccine.2009.07.060 19654064 PMC3571653

[14] Lai CH Tang N Jan JT Huang MH Lu CY Chiang BL Use of recombinant flagellin in oil-in-water emulsions enhances hemagglutinin-specific mucosal IgA production and IL-17 secreting T cells against H5N1 avian influenza virus infection. Vaccine (2015) 33:4321–9. 10.1016/j.vaccine.2015.03.082 25858857

[15] Wegmann F Gartlan KH Harandi AM Brinckmann SA Coccia M Hillson WR Polyethyleneimine is a potent mucosal adjuvant for viral glycoprotein antigens. Nat Biotechnol (2012) 30:883–8. 10.1038/nbt.2344 22922673 PMC3496939

[16] Yang K Whalen BJ Tirabassi RS Selin LK Levchenko TS Torchilin VP A DNA vaccine prime followed by a liposome-encapsulated protein boost confers enhanced mucosal immune responses and protection. J Immunol (2008) 180:6159–67. 10.4049/jimmunol.180.9.6159 18424737 PMC3633597

[17] Kunisawa J Kurashima Y Kiyono H. Gut-associated lymphoid tissues for the development of oral vaccines. Adv Drug Deliv Rev (2012) 64:523–30. 10.1016/j.addr.2011.07.003 21827802

[18] Asanuma H Fujihashi K Miyakoshi T Yoshikawa T Fujita-Yamaguchi Y Kojima N Long- and short-time immunological memory in different strains of mice given nasally an adjuvant-combined nasal influenza vaccine. Vaccine (2007) 25:6975–80. 10.1016/j.vaccine.2007.06.060 17716790

[19] Belshe RB Edwards KM Vesikari T Black SV Walker RE Hultquist M Live attenuated versus inactivated influenza vaccine in infants and young children. N Engl J Med (2007) 356:685–96. 10.1056/NEJMoa065368 17301299

[20] Gruber WC Hinson HP Holland KL Thompson JM Reed GW Wright PF. Comparative trial of large-particle aerosol and nose drop administration of live attenuated influenza vaccines. J Infect Dis (1993) 168:1282–5. 10.1093/infdis/168.5.1282 8228364

[21] Harden KA Narden RM Response of patients offered influenza vaccination by injection and by nasal insufflation. Br Med J (1977) 1:686 10.1136/bmj.1.6062.686 PMC1605514 843871

[22] Barria MI Garrido JL Stein C Scher E Ge YC Engel SM Localized mucosal response to intranasal live attenuated influenza vaccine in adults. J Infect Dis (2013) 207:115–24. 10.1093/infdis/jis641 23087433 PMC3571238

[23] Ross TM Mahmood K Crevar CJ Schneider-Ohrum K Heaton PM Bright RA. A trivalent virus-like particle vaccine elicits protective immune responses against seasonal influenza strains in mice and ferrets. PLoS One (2009) 4:e6032. 10.1371/journal.pone.0006032 19554101 PMC2698286

[24] Wareing MD Tannock GA. Route of administration is the prime determinant of IgA and IgG2a responses in the respiratory tract of mice to the cold-adapted live attenuated influenza A donor strain A/Leningrad/134/17/57. Vaccine (2003) 21:3097–100. 10.1016/S0264-410X(03)00262-7 12804835

[25] Barroso SPC Nico D Nascimento D Santos ACV Couceiro JNSS Bozza FA Intranasal immunization with pressure inactivated avian influenza elicits cellular and humoral responses in mice. PLoS One (2015) 10:e0128785. 10.1371/journal.pone.0128785 26056825 PMC4461174

[26] Sun K Ye J Perez DR Metzger DW. Seasonal FluMist vaccination induces cross-reactive T cell immunity against H1N1 (2009) influenza and secondary bacterial infections. J Immunol (2011) 186:987–93. 10.4049/jimmunol.1002664 21160043

[27] Chaung HC Cheng LT Hung LH Tsai PC Skountzou I Wang B *Salmonella* flagellin enhances mucosal immunity of avian influenza vaccine in chickens. Vet Microbiol (2012) 157:69–77. 10.1016/j.vetmic.2011.12.014 22226542

[28] Doria-Rose NA Haigwood NL. DNA vaccine strategies: candidates for immune modulation and immunization regimens. Methods (2003) 31:207–16. 10.1016/S1046-2023(03)00135-X 14511953

[29] Jiang YH Li M Zhang ZR Gong T Sun X. Enhancement of nasal HIV vaccination with adenoviral vector-based nanocomplexes using mucoadhesive and DC-targeting adjuvants. Pharm Res (2014) 31:2748–61. 10.1007/s11095-014-1372-9 24792827

[30] Hong SH Byun YH Nguyen CT Kim SY Seong BL Park S Intranasal administration of a flagellin-adjuvanted inactivated influenza vaccine enhances mucosal immune responses to protect mice against lethal infection. Vaccine (2012) 30:466–74. 10.1016/j.vaccine.2011.10.058 22051136

[31] Sheppard NC Brinckmann SA Gartlan KH Puthia M Svanborg C Krashias G Polyethyleneimine is a potent systemic adjuvant for glycoprotein antigens. Int Immunol (2014) 26:531–8. 10.1093/intimm/dxu055 24844701

[32] Myc A Kukowska-Latallo JF Bielinska AU Cao P Myc PP Janczak K Development of immune response that protects mice from viral pneumonitis after a single intranasal immunization with influenza A virus and nanoemulsion. Vaccine (2003) 21:3801–14. 10.1016/S0264-410X(03)00381-5 12922114

[33] Kirschner M Monrose V Paluch M Techodamrongsin N Rethwilm A Moore JP The production of cleaved, trimeric human immunodeficiency virus type 1 (HIV-1) envelope glycoprotein vaccine antigens and infectious pseudoviruses using linear polyethylenimine as a transfection reagent. Protein Expr Purif (2006) 48:61–8. 10.1016/j.pep.2006.02.017 16600625

[34] Honko AN Mizel SB. Mucosal administration of flagellin induces innate immunity in the mouse lung. Infect Immun (2004) 72:6676–9. 10.1128/IAI.72.11.6676-6679.2004 15501801 PMC523048

[35] Singh SM Alkie TN Nagy É Kulkarni RR Hodgins DC Sharif S. Delivery of an inactivated avian influenza virus vaccine adjuvanted with poly(D,L-lactic-co-glycolic acid) encapsulated CpG ODN induces protective immune responses in chickens. Vaccine (2016) 34:4807–13. 10.1016/j.vaccine.2016.08.009 27543454

[36] Garcon N Morel S Didierlaurent A Descamps D Wettendorff M Van Mechelen M. Development of an AS04-adjuvanted HPV vaccine with the adjuvant system approach. BioDrugs (2011) 25:217–26. 10.2165/11591760-000000000-00000 21815697

[37] Khatun S Hussain SMA Chowdhury S Ferdous J Hossain F Begum SR Safety and immunogenicity profile of human papillomavirus-16/18 AS04 adjuvant cervical cancer vaccine: a randomized controlled trial in healthy adolescent girls of Bangladesh. Jpn J Clin Oncol (2012) 42:36–41. 10.1093/jjco/hyr173 22194637 PMC3244935

[38] McKeage K Romanowski B AS04-adjuvanted human papillomavirus (HPV) types 16 and 18 vaccine (cervarix®) a review of its use in the prevention of premalignant cervical lesions and cervical cancer causally related to certain oncogenic HPV types. Drugs (2011) 71:465–88. 10.2165/11206820-000000000-00000 21395359

[39] Frenck RW Belshe R Brady RC Winokur PL Campbell JD Treanor J Comparison of the immunogenicity and safety of a split-virion, inactivated, trivalent influenza vaccine (Fluzone®) administered by intradermal and intramuscular route in healthy adults. Vaccine (2011) 29:5666–74. 10.1016/j.vaccine.2011.06.010 21699951 PMC3150501

