# Corrigendum: Mucosal and Systemic Immune Responses to Influenza H7N9 Antigen HA1–2 Co-Delivered Intranasally With Flagellin or Polyethyleneimine in Mice and Chickens

**DOI:** 10.3389/fimmu.2018.01846

**Published:** 2018-08-13

**Authors:** Li Song, Dan Xiong, Hongqin Song, Lili Wu, Meihua Zhang, Xilong Kang, Zhiming Pan, Xinan Jiao

**Affiliations:** ^1^Jiangsu Co-Innovation Center for Prevention and Control of Important Animal Infectious Diseases and Zoonoses, Yangzhou University, Yangzhou, China; ^2^Jiangsu Key Laboratory of Zoonosis, Yangzhou University, Yangzhou, China; ^3^Key Laboratory of Prevention and Control of Biological Hazard Factors (Animal Origin) for Agrifood Safety and Quality, Ministry of Agriculture of China, Yangzhou University, Yangzhou, China; ^4^Joint International Research Laboratory of Agriculture and Agri-Product Safety of the Ministry of Education, Yangzhou University, Yangzhou, China

**Keywords:** avian influenza A (H7N9) virus, hemagglutinin globular head, flagellin, polyethyleneimine, mucosal subunit vaccine

In the published article, there was an error in affiliations 1 and 4 as Yangzhou University was not included. The authors apologize for this error and state that this does not change the scientific conclusions of the article in any way.

The original article has been updated.

## Conflict of Interest Statement

The authors declare that the research was conducted in the absence of any commercial or financial relationships that could be construed as a potential conflict of interest.

