# Supplementary material for: Mucosal and Systemic Immune Responses to Influenza H7N9 Antigen HA1–2 Co-Delivered Intranasally with Flagellin or Polyethyleneimine in Mice and Chickens
Source: Front Immunol. 2017 Apr 5;8:326. doi: 10.3389/fimmu.2017.00326 (PMC5380672; doi:10.3389/fimmu.2017.00326)
Supplement: Supplementary file 1 [file Data_Sheet_1.DOCX]

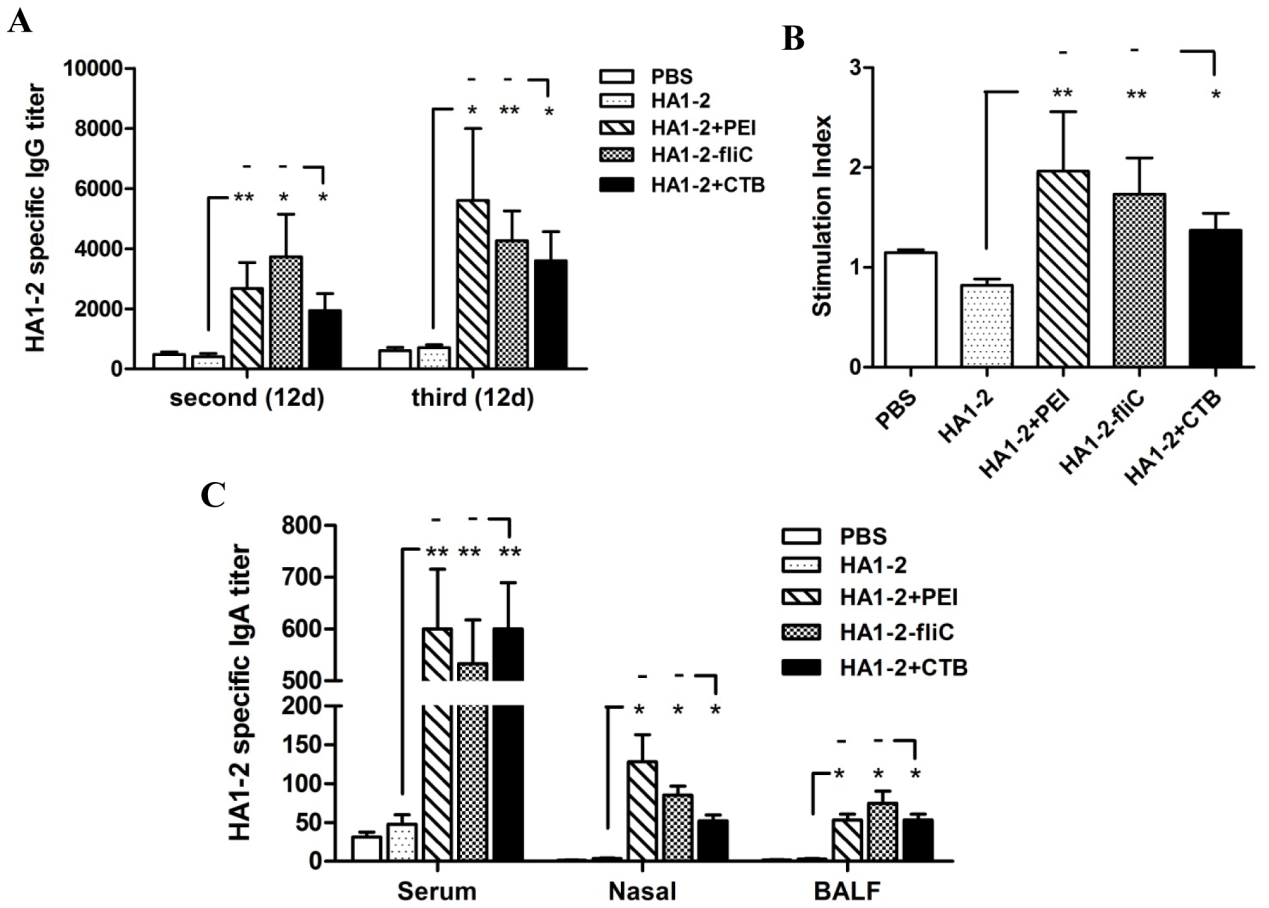


**Supplementary Figure 1**. **Immune response determination in chickens.** (A) Humoral immune response. All chickens were bled from the wing vein 12 days after the second and third immunization. Serum samples separated from whole blood were analyzed by ELISA to determine HA1-2-specific IgG titers. (B) Cellular immune response. At two weeks after the third immunization, chickens (*n* = 6) were bled and sacrificed. PBMCs were prepared from chickens per group and the stimulation index (SI) of cells in response to purified HA1-2 (10 μg/ml) protein was calculated based on cell proliferation, SI = (OD_450_ − OD_690_ of antigen-treated cells)/(OD_450_ − OD_690_ of untreated cells). (C) Mucosal immune response. Chickens (*n* = 6) were bled at two weeks after the third immunization, and the nasal wash and BALF were obtained by washing the organs three times with 0.5 or 1.0 ml sterile PBS, respectively, and IgA titers were determined by ELISA.
